# ﻿*Melhania orriana* (Malvaceae, Dombeyoideae), a new species from Somalia

**DOI:** 10.3897/phytokeys.263.163436

**Published:** 2025-09-24

**Authors:** Laurence J. Dorr

**Affiliations:** 1 Department of Botany, MRC-166, National Museum of Natural History, Smithsonian Institution, P.O. Box 37012, Washington, D.C. 20013–7012, USA National Museum of Natural History, Smithsonian Institution Washington United States of America

**Keywords:** Dombeyoideae, Malvaceae, *
Melhania
*, Somalia

## Abstract

*Melhania orriana* Dorr, **sp. nov.**, is described and illustrated. It is known only from central and southern Somalia at low elevations. The new species is morphologically similar to *M. stipulosa* J.R.I. Wood, which was described from Yemen and occurs in Ethiopia and Somalia at high elevations.

## ﻿Introduction

As circumscribed by Dorr and Wurdack (2021), *Melhania* Forssk. (Malvaceae, Dombeyoideae) is a genus of ca. 75 species found in St. Helena, continental Africa, the Arabian Peninsula, Madagascar, southern Asia (Pakistan, India, Myanmar, China), Malesia (Lesser Sunda Islands, New Guinea), and Australia. Geographically, it is the most widespread genus in the subfamily. Most species occur in Africa and Madagascar, and centers of species diversity appear to be in northeastern Africa and southwestern Madagascar. In northeastern Africa, 15 species are reported for Somalia (Thulin 1999; Baldesi et al. 2022); 17 species, including at least one undescribed species, are known from neighboring Ethiopia and Eritrea (Vollesen 1995); and 13 species are recognized as occurring in tropical East Africa (Kenya, Tanzania, and Uganda) (Cheek and Dorr 2007).

While preparing the account of *Melhania* for the “Flora of Tropical East Africa” (Cheek and Dorr 2007), several sheets of *Melhania* identified as *M. coriacea* Chiov., including one cited in the “Flora of Somalia” (Thulin 1999), were set aside because they appeared to be distinct from the type of that species. Further investigation confirmed that they represent an undescribed species, which is described below.

## ﻿Taxonomic summary

### ﻿ Melhania orriana

Taxon classificationPlantaeMalvalesMalvaceae

Dorr sp. nov.

2C0E9F8B-1058-5091-90B2-0E3914E824EE

urn:lsid:ipni.org:names:77369657-1

Fig. 1

#### Diagnosis.

*Melhania orriana*, with its elliptic-oblong, strap-shaped leaves and long, persistent stipules, is superficially similar to *M. stipulosa* J.R.I. Wood, but its leaf blade margin is crenulate or obscurely to conspicuously sinuate (versus entire and only obscurely sinuate toward the apex), its inflorescences are 2–3(–4)-flowered (versus solitary), and its epicalyx bracts in fruit are ovate with truncate to shallowly cordate bases (versus ovate with noticeably cordate bases) and dull pink (versus yellow green).

**Figure 1.**
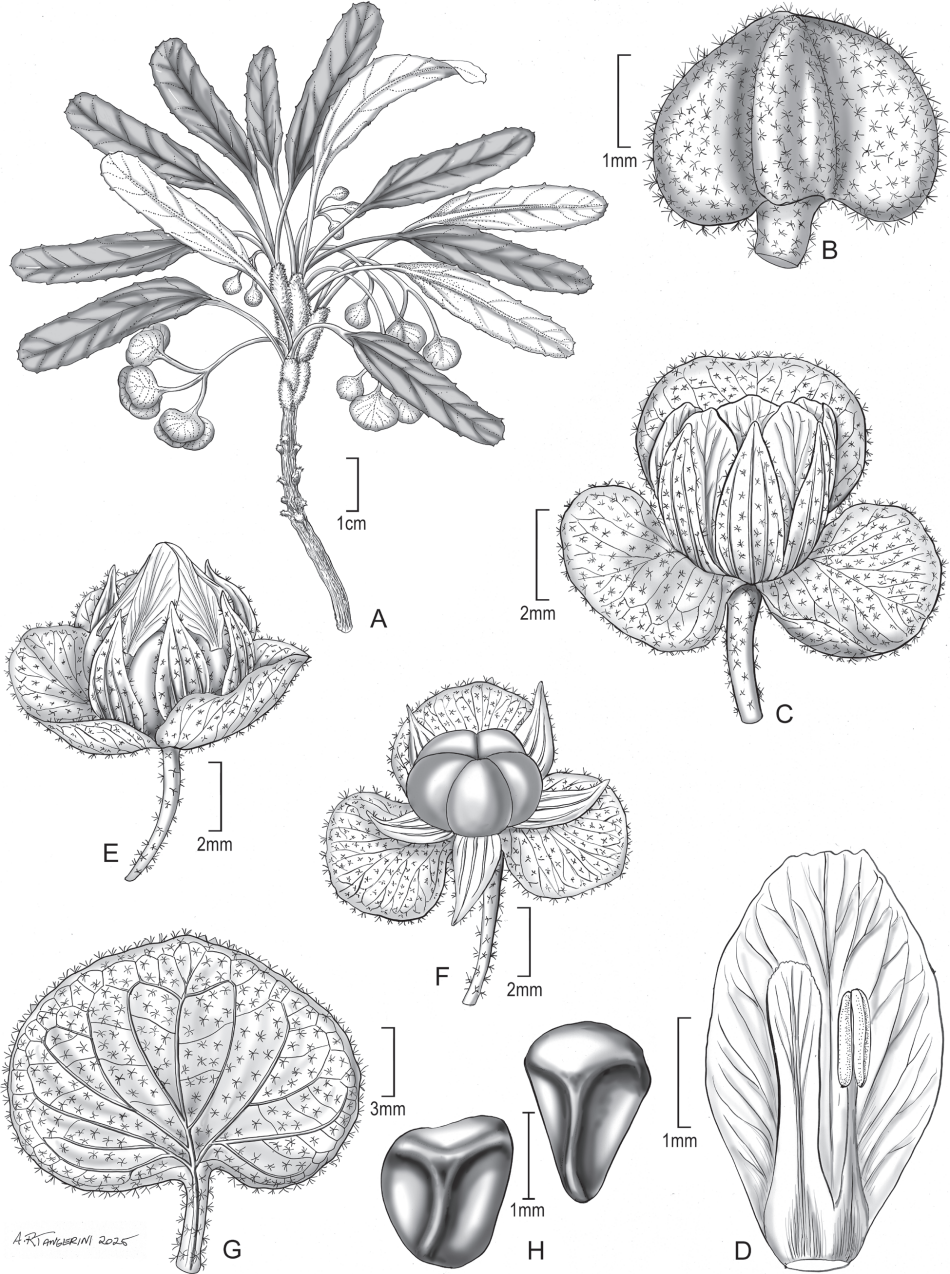
*Melhania orriana* Dorr, sp. nov. A. Habit; B. Flower bud; C. Flower at anthesis showing epicalyx lobes, sepals, and petals; D. Petal, staminode, and anther, the last two briefly joined at base; E. Flower in fruit showing the persistent corolla; F. Flower in fruit with petals removed showing 5-loculed ovary; G. Epicalyx bract in fruit; H. Seeds (Vouchers: A–D from *Thulin & Abdi M. Dahir 6445* (K); E–H from *Thulin & Abdi M. Dahir 6748* (K)).

#### Type.

Somalia • Hiran: 2.5 km on road between Ceel Baraf and Aadan Yabaal, 3°31'N, 45°46'E, 125 m elev., 23 May 1989 (fl, fr), *M. Thulin & Abdi M. Dahir 6445* (holotype: K!; isotype: UPS [V-048644]-n.v.).

#### Description.

Perennial herbs or shrublets, 6–8 cm tall; stems decumbent or ascending, unfurled leaves whitish pubescent; taproots woody, small, slender, to ca. 6 cm long. Leaves clustered near the tips of the branches, internodes very short, leaf blades elliptic-oblong (strap shaped), 2.2–5 × 0.4–1.1 cm, apex broadly acute, almost truncate, shortly apiculate, base attenuate to cuneate, margin crenulate or obscurely to conspicuously sinuate, often appearing cinereous from above before leaf is fully mature, discolorous, yellow-green, and densely stellate-tomentose above; cinereous and densely stellate-tomentose below with some stellate hairs with dark centers; petioles slender, thickened apically, 0.6–1.5(–2) cm long, densely stellate-tomentose, rays spreading and some hairs stalked; stipules acicular or filiform, 5–10 mm long, sparingly stellate pubescent, often reddish turning black, persistent. Inflorescences axillary, 2–3(–4)-flowered cymes; peduncles slender, almost filiform, to 1.5 cm long at anthesis and to 2.5 cm long in fruit, stellate pubescent with rays spreading and some hairs stalked; pedicels filiform, 2–4 mm long, stellate pubescent with rays spreading and some hairs stalked. Epicalyx bracts 3, in bud ovate, base truncate to shallowly cordate, apex rounded, ca. 3 × 4 mm (height × width) and cinereous-tomentose, in fruit more broadly accrescent, 9–12 × 12–19 mm (height × width) and stellate-puberulent, membranous, and dull pink. Sepals 5, lanceolate, almost free, at anthesis ca. 4 × 1.5 mm, stellate pubescent, in fruit accrescent, ca. 5 × 2 mm, membranous, stellate pubescent. Petals 5, narrowly obovate, slightly oblique, 4–5 × 3 mm, pale yellow, persisting in fruit. Stamens 5, ca. 3 mm long, anthers lanceolate; staminodes 5, ligulate, ca. 3–3.5 mm long (slightly exceeding stamens in length); stamens and staminodes briefly joined into a tube at base. Ovary globose, 5-locular, ca. 4 × 4 mm, stellate pubescent; style 1; stigma lobes 5. Capsules eventually nutant, enclosed by the accrescent epicalyx bracts, globose, 5–7 × 5–6 mm, stellate pubescent, 5-valved, each locule with 2 seeds; seeds obtrigonous, ca. 2 × 1.5 mm, brown, smooth or inconspicuously tuberculate.

#### Etymology.

The specific epithet honors Blair D. Orr, who, from 1982 to 1983 while employed by Interchurch Response, Luuq, Somalia, was a project and field forester for a forestry program in eight refugee camps. Subsequently, he entered academia, and following four years teaching at the University of the South, Sewanee, Tennessee, USA, he was for several decades a professor of Forest Resources & Environmental Science and director of Peace Corps Programs at Michigan Technological University, Houghton, Michigan, USA, until his retirement in 2022.

#### Distribution and habitat.

Endemic to central and southern Somalia, where it is found on deep sandy soil, including on fixed dunes of orange or white sand in Acacia-Dichrostachys bushland; 50–125 m elev.

#### Additional material examined.

Somalia • **Banaadir**: Mogadishu, N edge of town near Guled Hotel, 1 Feb 1982 (fr), *M. Thulin 4149* (K). • **Middle Shabelle**: Shabeellaha Dhexe Region, 179 km NE of Mogadishu, on road to Harardere, 90 m elev., 25 Sep 1985 (fl), *J.J. Lavranos & S. Carter* 23277 (K [K005052981], MO); • 16 km on road between Cadale and Ceeldheer (El Dere), 50 m elev., 2°55'N, 46°18'E, 30 May 1989 (fl), *M. Thulin & Abdi M. Dahir 6748* (K, UPS [V-048947]-n.v.).

#### Discussion.

In addition to the morphological differences mentioned in the diagnosis, *Melhania orriana* differs from *M. stipulosa* in its distribution and ecology. While the new species evidently is restricted to sandy soil in *Acacia–Dichrostachys* bushland in central and southern Somalia at low elevations (50–125 m elev.), *M. stipulosa*, described from Yemen (Wood 1984), occurs in well-drained stony soil on exposed ridges and open bushland in the highlands (1200–2500 m elev.) of southwestern Arabia as well as in the highlands of Ethiopia and Somalia. Although the infrageneric classification of *Melhania* needs revision (see Cheek and Dorr 2007), both species, with their conspicuous ovate epicalyx bracts that are accrescent and membranous in fruit, would fit in M. subgen. Hymenonephros K. Schum. *Melhania orriana* also differs from the superficially similar *M. muricata* Balf. f., described from Socotra but known also from the Arabian Peninsula, Kenya, and Somalia. *Melhania muricata* has shorter stipules and pedicels, and its seeds are conspicuously tuberculate.

Thulin (1999) cited Thulin & Abdi M. Dahir 6445, selected here as the type of *Melhania orriana*, as one of the collections he identified as *M. coriacea*. However, these two species have very different habits. The latter, unlike the former, is a larger (6–15 cm tall), detritus-collecting subshrub. As circumscribed by Thulin (1999), *M. coriacea* was very variable in indumentum and in the size and shape of leaves and epicalyx bracts. Segregating the misplaced material as *M. orriana* does make both species much more homogeneous.

Thulin (1999) also noted that Chiovenda (1929) originally stated that *Melhania coriacea* was unique within the genus in having a 3-carpellate ovary and consequently proposed a new subgenus, M. subgen. Trigynella Chiov., to accommodate it. The material of *M. coriacea* examined in this study is 5-carpellate, as are all other members of the genus. One can only assume, as did Thulin (1999), that Chiovenda misinterpreted the holotype, the only specimen known to him.

## Supplementary Material

XML Treatment for Melhania orriana

## ﻿Additional information

### Use of AI

No use of AI was reported.

### Author ORCIDs

Laurence J. Dorr https://orcid.org/0000-0001-7157-363X

### Data availability

All of the data that support the findings of this study are available in the main text.
